# Diversity of *Trametes* (Polyporales, Basidiomycota) in tropical Benin and description of new species *Trametes parvispora*

**DOI:** 10.3897/mycokeys.65.47574

**Published:** 2020-03-10

**Authors:** Boris Armel Olou, Franz-Sebastian Krah, Meike Piepenbring, Nourou Soulemane Yorou, Ewald Langer

**Affiliations:** 1 Department of Ecology, Universität Kassel, Heinrich-Plett-Str. 40, Kassel, Germany; 2 Research Unit Tropical Mycology and Plant-Soil Fungi Interactions (MyTIPS), University of Parakou BP 123, Parakou, Benin; 3 Laboratory of Applied Ecology, University of Abomey-Calavi (LEA/UAC), Benin; 4 Animal Ecology, Department of Ecology, Faculty of Biology, Philipps-Universität Marburg, Marburg, Germany; 5 Bavarian Forest National Park, Freyunger Str. 2, 94481 Grafenau, Germany; 6 Department of Mycology, Biologicum, Goethe Universität, Max-von-Laue-Str. 13, 60438 Frankfurt am Main, Germany

**Keywords:** Africa, morphology, new taxa, phylogeny, Polyporales, taxonomy, tropics, white rot

## Abstract

*Trametes* is a globally distributed genus of white-rot polypores and well sampled in temperate and boreal areas. However, the diversity, taxonomy, and phylogenetic positions of *Trametes* spp. are poorly known in tropical Africa. This study aims at documenting the diversity of *Trametes* species in Benin (tropical Africa) and their phylogenetic positions with a focus on the *T. elegans* species complex. Therefore, we collected specimens of *Trametes* from different forest types across Benin. To infer phylogenetic relationships between *Trametes* species, we investigated sequences of five gene regions and added available sequences from GenBank. Using Maximum likelihood and Bayesian phylogeny inference methods, we found eight supported species clades. For the *T. elegans* species complex, we re-establish the name *Trametes palisotii* for species previously known as *T. elegans* in tropical Africa. Furthermore, we propose *Trametes parvispora* as a species new to science and provide the description of this species. Our molecular phylogeny of *Trametes* with a focus on tropical Benin contributes to taxonomic clarity of an important wood-decay fungal genus, which is the basis for biodiversity assessments of *Trametes* in the tropics.

## Introduction

The genus *Trametes* Fr. (Polyporales, Basidiomycota) consists of wood-decay fungi with a distribution covering all continents and all major climatic zones (Gilbertson and Ryvarden 1987; Ryvarden 1991). Species of *Trametes* are characterized by a combination of a pileate basidioma, a poroid hymenophore, a trimitic hyphal system, and non-amyloid, thin-walled basidiospores (Gilbertson and Ryvarden 1987). They are saprotrophs causing white rot during the decay of woody substrates (Wong and Wilkes 1988). Species of the genus *Trametes* have a long ethnomycological history as medicinal fungi in many cultures (Cui et al. 2011; Ss and Pandey 2012; Ueitele et al. 2017) and some species are studied in the context of cancer research (Zmitrovich et al. 2012; Cruz et al. 2016; Blagodatski et al. 2018). Despite the global-scale distribution, importance for wood decomposition, and medicinal properties, the taxonomic and phylogenetic knowledge of *Trametes* spp. worldwide is still incomplete (Carlson et al. 2014).

Since the first formal description of the genus *Trametes* by Fries (1835), based on the type species *Trametes suaveolens* (L.) Fr., the concept of this genus was interpreted in different ways, resulting in different numbers of species attributed to the genus (Karsten 1881; Murrill 1905; Kavina and Pilát 1936; Kotlaba and Pouzar 1957; Gilbertson and Ryvarden 1987; Corner 1989). Recently, based on phylogenetic analyses, the concept of *Trametes* was re-delimited and circumscribed (Justo and Hibbett 2011). Here, we apply the broad concept of *Trametes* as proposed by Justo and Hibbett (2011). This concept includes in addition to species of *Trametes* sensu stricto, species of *Artolenzites* Falck, *Coriolopsis* Murrill, *Lenzites* Fr., and *Pycnoporus* P. Karst.

**Figure 1.**
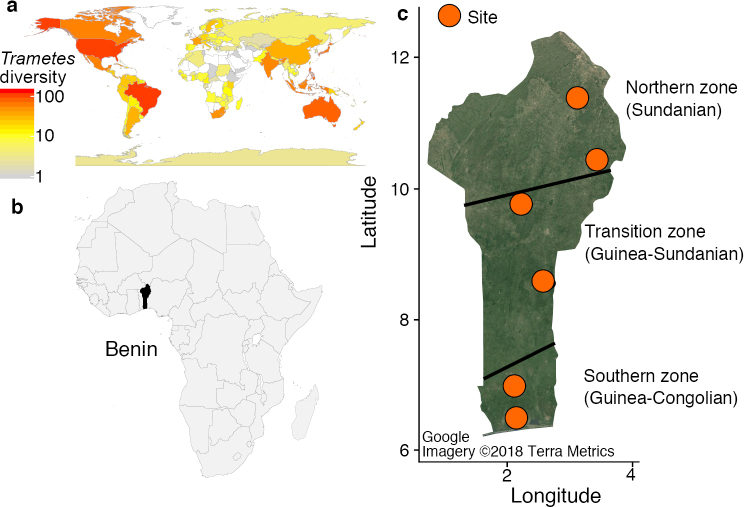
**a** Observations of *Trametes* spp. retrieved from MyCoPortal and GBIF, based on herbarium specimens and citizen science observations **b** The study area (Benin) in the western part of Africa (highlighted in black) **c** Locations of the sampling sites within macroclimatic zones, which are delimited by black lines. The circles in orange indicate respectively from bottom to top the sampling sites: dry dense forest of Pahou, dense semi-deciduous forest of Lama, woodlands of Kilibo, woodlands of Ouémé Superieur, Trois Rivières woodland, and savanna ecosystems of the national park W.

Previous studies on *Trametes* spp. mainly concentrated on specimens from temperate and boreal regions (David 1967; Gilbertson and Ryvarden 1987; Hattori 2005; Tomšovský et al. 2006; Pieri and Rivoire 2007; Ryvarden et al. 2009; Gomes-Silva et al. 2010; Hattori and Sotome 2013), and thus most *Trametes* spp. have been described from these regions. By contrast, little is known on *Trametes* spp. in tropical Africa (Fig. 1A), and most known specimens of *Trametes* spp. from this area are missing in most phylogenetic analyses.

For Benin, seven species of *Trametes*, namely *T. cingulata* Berk., *T. elegans* (Spreng.) Fr., *T. flavida* (Lév.) Zmitr., Wasser & Ezhov (cited as *Leiotrametes flavida*), *T. polyzona* (Pers.) Justo, *T. sanguinea* (L.) Lloyd (cited as *Pycnoporus sanguineus*), and *T. socotrana* Cooke were reported by Olou et al. (2019). Taking a closer look at these species, we noticed that sequence data are lacking for specimens from tropical Africa and that the knowledge on taxonomical and phylogenetic placements is incomplete.

Additional to these known species in Benin, we recently found a putatively new species of *Trametes* (Olou et al. 2019), but morphological and phylogenetic analyses were outstanding. In the same study, we reported the occurrence of *T. elegans* in Benin.

*Trametes elegans* was found to be a species complex and has therefore recently been split into three distinct species, namely *T. aesculi* (Fr.) Justo, *T. elegans* s.str., and *T. repanda* (Pers.) Justo (Carlson et al. 2014). However, this study did not include tropical African specimens although *T. elegans* exists in this area.

Our study thus aims to report the diversity of *Trametes* species in Benin and their phylogenetic positions, with a focus on a new species of *Trametes* and the *T. elegans* species complex.

## Material and methods

### Specimens sampling and preservation

A total of 37 specimens of *Trametes* were collected in three different macroclimatic zones and different forests of Benin (Fig. 1A, C) from July to September in 2017 (Olou et al. 2019) and in 2018 (another series of surveys). Small pieces of fresh fruit bodies were placed in plastic bags half-filled with silica gel for DNA extraction. The rest of fruit bodies were air- or oven-dried at 45–50 °C for 1–2 days depending on the consistency of the fruit body. The dried fruit bodies were then preserved in plastic bags for morphological investigation. Specimens are deposited at the mycological herbaria of the University of Parakou (UNIPAR; Thiers 2019) and the University of Kassel (KAS).

### DNA extraction, amplification, sequencing and alignment

**DNA extraction.** Genomic DNA of all specimens classified into nine morphotypes was extracted using the microwave DNA extraction method (Dörnte and Kües 2013) or the NucleoSpin Plant II DNA extraction kit (Macherey, Nagel, Germany).

**Amplifications and sequencing.** The extracted genomic DNA was amplified targeting two nuclear ribosomal DNA (*nr*DNA) regions, internal transcribed spacer (ITS) and ribosomal large subunit-coding DNA (28S rRNA) for all specimens. Additionally, three protein-coding genes, RNA polymerase II largest subunit (RPB1), RNA polymerase II second largest subunit (RPB2), and translation elongation factor 1-alpha (TEF1) were amplified for specimens forming part of the *T. elegans* species complex and specimens of *Trametes* sp. The amplification of the 5.8S rRNA gene region, including ITS were performed in Mastercycler nexus gradient (Eppendorf, Germany), using the primer pair ITS-1F/ITS4 (White et al. 1990; Gardes and Bruns 1993). The Polymerase Chain Reaction (PCR) procedure for the ITS region, was as follows: initial denaturation at 95 °C for 3 min, followed by 35 cycles at 95 °C for 30 s, 52 °C for 30 s and 68 °C for 1 min, and a final extension at 68 °C for 3 min. Amplifications of LSU and three protein-coding genes were performed in 96-well TGradient Thermocycler (Biometra, Göttingen, Germany). PCR procedure for amplifying partial LSU rDNA using the primer pair LR0R/LR5 (Vilgalys and Hester 1990) approximately 964 bp differed to the ITS only by the annealing temperature (55 °C instead of 52 °C) and increased cycle extension time (90 s per cycle). The primer pairs EF1-983F/EF1-1567R (Rehner and Buckley 2005), RPB1-Af/RPB1-Cr (Stiller and Hall 1997; Matheny et al. 2002), and RPB2-b6F/RPB2-b7.1R (Liu et al. 1999; Matheny 2005) were used to amplify approximately 500 bp of TEF1, 1000 bp of RPB1, and 800 bp of RPB2. To amplify the protein-coding genes RPB1 and RPB2, the touchdown PCR protocol following Justo and Hibbett (2011) was used. PCR products were checked on 1% agarose gel stained with GelRed fluorescence dye (Biotium, Hayward, California, USA) in the Transilluminator Biometra Ti5 equipped with BioDocAnalyze software (Biometra GmbH, Göttingen, Germany). They were further cleaned up with QIAquick PCR Purification Kit according to manufacturer’s instructions (QIAGEN GmbH, Hilden, Germany). Thereafter, Sanger sequenced at GATC Biotech in Germany.

**Figure 2.**
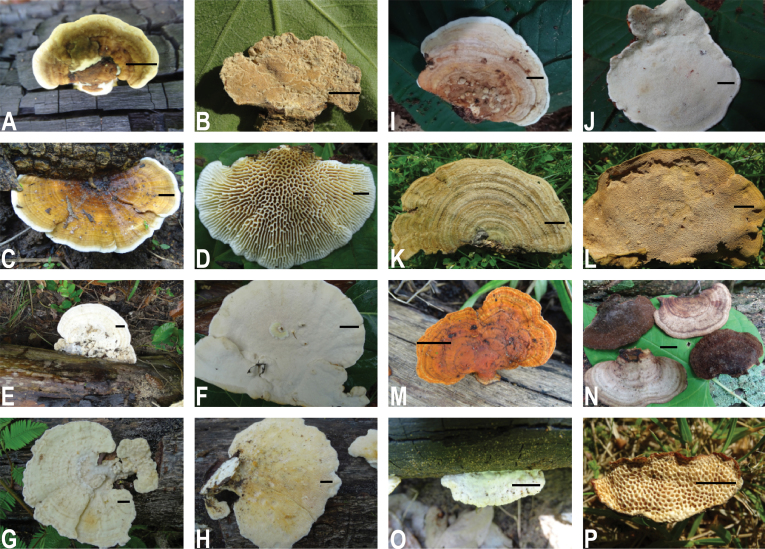
Macromorphology of *Trametes* species in Benin and specimen numbers in parentheses. **A***Trametes cingulata***B** hymenophore of *Trametes cingulata* (10) **C***Trametes flavida***D** hymenophore of *Trametes flavida* (05) **E***Trametes lactinea***F** hymenophore of *Trametes lactinea* (01) **G***Trametes palisotii***H** hymenophore of *Trametes palisotii* (04) **I***Trametes parvispora***J** hymenophore of *Trametes parvispora* (04) **K***Trametes polyzona***L** hymenophore of *Trametes polyzona* (06) **M***Trametes sanguinea* (04) **N**Trametes aff. versicolor (01) **O***Trametes socotrana***P** hymenophore of *Trametes socotrana* (02). Scale bar corresponds to 1cm except in **E**, **F** where it corresponds to 2 cm.

At least one sequence per specimen was generated for each morphotype except for the morphotype named Trametes aff. versicolor (Fig. 2N; Suppl. material 1). All newly generated sequences composed of 25 ITS, 20 LSU, two RPB1, four RPB2, and three TEF1 were deposited in GenBank (for accession numbers, see Table 1).

**Table 1.** Taxa names with collection details and GenBank accession numbers of all sequences of *Trametes* spp.

Species name	Voucher or strain	Origin	GenBank N°					Reference
			ITS	LSU	RPB1	RPB2	TEF1	
*Dentocorticium sulphurellum*	FP11801		JN165018	JN164815	JN164841	JN164876		Justo and Hibbett 2011
*Lopharia cinerascens*	FP105043sp	USA: Mississippi	JN165019	JN164813	JN164840	JN164874		Justo and Hibbett 2011
*T. aesculi* (*T. elegans* species complex)	HHB4626sp	USA	JN164950		KF573173	KF573134	KF573083	Justo and Hibbett 2011, Carlson et al. 2014
	FP105679sp	USA/Georgia	JN164944	JN164799	JN164833	JN164861	JN164899	
	HHB6551	USA/Florida	JN164938		KF573172	KF573136	KF573082	
	FP105038sp	USA: Mississippi	JN164951		KF573174	KF573135	KF573081	
*T. betulina* (*Lenzites betulinus*)	HHB9942sp	USA	JN164983	JN164794		JN164860		Justo and Hibbett 2011
	Dai6847		KC848305	KC848390				unpublished
*T. cingulata*	MUCL:40167	Malawi	JN645075					Welti et al. 2012
	Dollinger 629	USA/Florida	KY264043					unpublished
	DMC814	Cameroon	KC589133	KC589159				unpublished
	**OAB0135**	**Benin**	**MK736973**					**this study**
	**OAB0117**	**Benin**	**MK736972**					
	**OAB0093**	**Benin**	**MK736970**					
	**OAB0114**	**Benin**	**MK736971**	**MK736950**				
	**OAB0161**	**Benin**	**MK736975**	**MK736951**				
	**OAB0155**	**Benin**	**MK736974**					
	**OAB0171**	**Benin**	**MK736976**	**MK736952**				
	**OAB0173**	**Benin**	**MK736977**	**MK736953**				
	**OAB0178**	**Benin**	**MK736978**	**MK736954**				
	**OAB0231**	**Benin**	**MK736979**	**MK736955**				
*T. cinnabarina* (cited as *Pycnoporus cinnabarinus*)	Dai 14386	China	KX880629	KX880667	KX880818	KX880854		unpublished
*T. coccinea* (cited as *Pycnoporus coccineus*)	Cui-7096		KC848330	KC848414				unpublished
*T. conchifer*	FP106793sp	USA/Mississippi	JN164924	JN164797	JN164823	JN164849		Justo and Hibbett 2011
*T. cubensis*	TJV93_213sp	USA/Mississippi	JN164923	JN164798	JN164834	JN164865		Justo and Hibbett 2011
	AJ177	USA: Florida	JN164905					
	UZ526_17	Malaysia	MF363158					unpublished
*T. ectypa*	FP103976sp	USA: FLorida	JN164961					Justo and Hibbett 2011
	FP106037T	USA	JN164929	JN164803	JN164824	JN164848		
*T. elegans* (*T. elegans* species complex)	PR1133	Puerto Rico	JN164937		KF573178	KF573139	KF573075	Justo and Hibbett 2011, Carlson et al. 2014
	FPRI10	Philippines	JN164973			KF573138	KF573074	
	FP150762	Belize	JN164928			KF573137	KF573076	
*T. flavida*	**OAB0047**	**Benin**	**MK736966**	**MK736946**				**this study**
	**OAB0090**	**Benin**	**MK736967**					
	**OAB0196**	**Benin**	**MK736968**	**MK736947**				
*T. flavida* (cited as *Leiotrametes flavida*)	DMC811	Cameroon	KC589130	KC589156				unpublished
	CBS 158.35		MH855616	MH867126				Vu et al. 2019
*T. gibbosa*	DMC815	Cameroon	KC589144	KC589164				unpublished
	L11664sp	England	JN164943	JN164800	JN164831	JN164859		Justo and Hibbett 2011
*T. hirsuta*	DMC341	Cameroon	KC589146	KC589166				unpublished
	RLG5133T	USA: New York	JN164941	JN164801	JN164829	JN164854		Justo and Hibbett 2011
*T. junipericola*	145295(O)		KC017758	KC017763				unpublished
*T. lactinea*	DMC346	Cameroon	KC589126	KC589152				unpublished
*T. lactinea (cited as Leiotrameteslactinea)*	CBS 109427	Taiwan	MH862825					Vu et al. 2019
*T. lactinea*	LIP:GUY09-110	French Guiana	JN645069					Welti et al. 2012
	Dai6865		KC848327	KC848411				unpublished
	**OAB0232**	**Benin**	**MK736983**	**MK736948**				**this study**
	BCC 33266	Thailand	GQ982888	GQ982881				unpublished
	Yuan5493		KC848320	KC848404				
*T. ljubarskyi*	Wei1653		KC848332	KC848416				unpublished
	Li286		KC848331	KC848415				
*T. maxima*	OH189sp	Venezuela	JN164957	JN164804	JN164816	JN164864		Justo and Hibbett 2011
*T. membranacea*	PRSC82	Puerto Rico	JN164945	JN164805	JN164832	JN164857		Justo and Hibbett 2011
*T. menziesii*	BRFM<FRA>:1368	Martinique	JN645103					Welti et al. 2012
	Dai6782		KC848289	KC848374				unpublished
*T. meyenii*		Philippines	JN164933		KF573179	KF573145		Justo and Hibbett 2011
*T. meyenii*	CBS:453.76	India	MH860991	MH872762				Vu et al. 2019
*T. ochracea*	HHB13445sp	USA/Michigan	JN164954	JN164812	JN164826	JN164852		Justo and Hibbett 2011
	Dai2005	China	KC848272	KC848357				unpublished
*T. palisotii* (*T. elegans* species complex)	**OAB0118**	**Benin**	**MK736980**	**MK736956**	**MK802884**	**MK802882**	**MK802886**	**this study**
	**OAB0153**	**Benin**	**MK736981**	**MK736957**	**MK802885**	**MK802883**	**MK802887**	
	**OAB0198**	**Benin**	**MK736982**	**MK736958**			**MK802888**	
*T. palisotii*	DMC360	Cameroon	KC589139	KC589160				unpublished
	DMC817	Cameroon	KC589142	KC589163				
	DMC816	Cameroon	KC589141	KC589162				
*T. parvispora*	**OAB0022**	**Benin**	**MK736989**	**MK736964**		**MN127965**		**this study**
	**OAB0023**	**Benin**	**MK736990**	**MK736965**		**MN127964**		
*T. pavonia*	FP103050sp	USA/Florida	JN164958	JN164806	JN164835	JN164862		Justo and Hibbett 2011
*T. polyzona*	DMC370	Cameroon	KC589125	KC589151				unpublished
	Cui 11040	China	KX880647	KX880689	KX880836	KR610849		
	BKW004	Ghana	JN164978	JN164790				Justo and Hibbett 2011
	**OAB0092**	**Benin**	**MK736984**	**MK736959**				**this study**
	**OAB0128**	**Benin**	**MK736985**	**MK736960**				
	**OAB0195**	**Benin**	**MK736986**	**MK736961**				
*T. pubescens*	FP101414sp	USA/Wisconsin	JN164963	JN164811	JN164827	JN164851		Justo and Hibbett 2011
*T. pucinea* (cited as *Pycnoporus puniceus*)	BCC26408	Thailand	FJ372685	FJ372707				unpublished
*T. punicea*	BCC27595		FJ372686	FJ372708				unpublished
*T. rependa* (*T. elegans* species complex)	FRI437T		JN164985		KF573177	KF573142	KF573080	Justo and Hibbett 2011, Carlson et al. 2014
	FPRI390	Philippines	JN164921		KF573175	KF573141	KF573077	
	OH271sp	Venezuela	JN164936		KF573176	KF573143	KF573079	
	M0138339	Papua New Guinea	KF573029			KF573140	KF573078	
*T. sanguinea*	**OAB0088**	**Benin**	**MK736969**	**MK736949**				**this study**
*T. sanguinea* (cited as *Pycnoporus sanguineus*)	PRSC95	Puerto Rico	JN164982	JN164795	JN164842	JN164858		Justo and Hibbett 2011
	BCC 36861	Thailand	GQ982885	GQ982878				unpublished
	8R_1_2	Thailand	FJ372672	FJ372694				
	CBS:614.73	Sri Lanka	MH860781	MH872513				
*T. socotrana*	BJFC12724	China	KC848313	KC848397				unpublished
	**OAB0131**	**Benin**	**MK736987**	**MK736962**				**this study**
	**OAB0162**	**Benin**	**MK736988**	**MK736963**				
*Trametes* sp. (cited as *Leiotrametes* sp.)	LIP:GUY08-156	French Guiana	JN645062					Welti et al. 2012
*Trametes* sp.	BC1	Finland	KT896651					Linnakoski et al. 2016
*Trametes* sp. (cited as *Leiotrametes* sp.)	LIP:GUY08-167	French Guiana	JN645063					Welti et al. 2012
*T. suaveolens*	FP102529sp	USA/Wisconsin	JN164966	JN164807	JN164828	JN164853		Justo and Hibbett 2011
	Dai 10729	China	JN048770	JN048789				unpublished
*T. versicolor*	FP135156sp	USA/New York	JN164919	JN164809	JN164825	JN164850		Justo and Hibbett 2011
*T. villosa*	FP71974R	USA/Tennessee	JN164969	JN164810	JN164830	JN164855		Justo and Hibbett 2011

The rows referring to sequences generated in this study are written in bold.

**Sequence alignment and phylogenetic analyses.** To place all the 25 generated ITS sequences of specimens of *Trametes* spp. in a phylogenetic context, we aligned them in addition to 66 ITS sequences retrieved from GenBank (Benson et al. 2011). Further, 48 LSU sequences were aligned with 20 LSU sequences generated here. For the *T. elegans* species complex, seven newly generated sequences of protein-coding genes were aligned in addition to sequences used by Carlson et al. (2014). Each marker was aligned separately using MAFFT version 7, with the algorithm L-INS-i (Katoh et al. 2017) and standard settings as default. The resulting multiple species alignments were slightly adjusted and trimmed at both ends a bit from incomplete sequences in Geneious 5.6.7 (Kearse et al. 2012). Eight different datasets were assembled for the phylogenetic analyses: (i) ITS dataset with 91 sequences of *Trametes* spp., (ii) combined ITS-LSU dataset with 91 sequences *Trametes* spp., (iii) combined RPB1-RPB2 dataset with 23 sequences of *Trametes* spp., (iv) ITS dataset with 17 sequences of *T. elegans* species complex, (v) RPB1 dataset with ten sequences of the *T. elegans* species complex, (vi) RPB2 dataset with 12 sequences of *T. elegans* species complex, (vii) TEF1 dataset with 14 sequences of *T. elegans* species complex, and (viii) combined dataset of four genes (ITS, RPB1, RPB2, TEF1) of *T. elegans* species complex. The combined datasets were concatenated using Geneious 5.6.7 (Kearse et al. 2012). For the phylogenetic analyses, the partitioning of the combined datasets of *Trametes* spp. was considered. *Lopharia cinerascens* (Schwein.) G. Cunn., and *Dentocorticium sulphurellum* (Peck) M.J. Larsen & Gilb., were chosen as the outgroup in all datasets (Justo and Hibbett 2011). Two phylogenetic tree inference methods, Maximum likelihood (ML) and Bayesian (BY) were performed in each dataset. The ML of all datasets were performed using RAxML 8.2.10 (Stamatakis 2014) and the BY of all individual genes and combined dataset of *T. elegans* species complex were performed using MrBayes 3.2.6 (Ronquist et al. 2012) at the Cipres Science Gateway V.3.3. (Miller et al. 2010). The BY of the partitioned datasets of *Trametes* spp. were run independently using MrBayes 3.2.7 (Ronquist et al. 2012). The parameters in BY inference were set as follows: lset applyto = (all), nst = 6, rates = invgamma, ngammacat = 4, sampling frequency = 1000, and the command “unlink” was used to unlink parameters across characters on partitioned datasets. Two independent Markov Chain Monte Carlo (MCMC) processes were run, each in 4 chains, for 5 million generations, and 0.2 fraction were discarded as burn-in. The Phylogenetic Tree Summarization (SumTrees) program within DendroPy 4.3.0. (Sukumaran and Holder 2010) was used to build the consensus tree with branch supports (posterior probabilities). Further, by using IQ-Tree (Trifinopoulos et al. 2016), we assigned the bootstrap values (BS) of ML to the consensus tree of BY. The resulting phylogenetic trees were inspected in FigTree v. 1.4.2 (Rambaut 2014). All sequence alignments and phylogenetic trees generated in the study were deposited in TreeBASE: http://purl.org/phylo/treebase/phylows/study/TB2:S24354. The topologies of the consensus trees obtained from BY are presented in all figures throughout the document. Posterior probabilities (PP) and bootstrap values (BS) on or below branches as followed (PP/BS).

### Microscopic analyses of specimens of the new species of *Trametes*

Macro-morphological descriptions were based on fresh and dried herbarium specimens. Microstructures are described using dried herbarium specimens. Fine sections through the basidiomata were prepared for observation using a razor blade under a stereomicroscope Leica EZ4 and mounted in 5% aqueous solution of potassium hydroxide (KOH) mixed with 1% aqueous solution of Phloxine. Melzer’s reagent (to test for dextrinoid or amyloid reactions), Cotton Blue (to test for cyanophilic reaction) were used and then examined at a magnification of 1000× using a Leica DM500 light microscope. Measurements were done with the software “Makroaufmaßprogramm” from Jens Rüdigs (https://ruedig.de/tmp/messprogramm.htm) and analysed with the software “Smaff” version 3.2 (Wilk 2012). In total, 135 basidiospores were measured from the sequenced specimen OAB0022 and additional examined specimen OAB0268. The basidiospore size is given as length and width of the spore. As measurements we present the mean with standard deviation and minimum and maximum values in parentheses (see below). The length (L), arithmetic average of all spore lengths, and the width (W), arithmetic average of all spore widths, were calculated. In addition, the ratio of length/width (Q) was calculated.

### Availability of data and materials

All alignments and phylogenetic trees generated in this study are available in TreeBASE under this link: http://purl.org/phylo/treebase/phylows/study/TB2:S24354. Newly generated sequences are available in GenBank, and the accession numbers are given in Table 1. Alignments, phylogenetic trees, and accession numbers of newly generated sequences will be public after the paper is published. Collected specimens are available at the mycological herbarium of the University of Parakou (UNIPAR). The new species was registered in mycoBank, and the registration number is given in the taxonomy section of this paper.

### Abbreviations

a.s.l. above sea level

BS Bootstrap values

BY Bayesian

ITS Internal Transcribed Spacer

KAS Mycological herbarium of the University of Kassel

L Length

LSU Large Subunit

MCMC Markov chain Monte Carlo

ML Maximum likelihood

*nr*DNA nuclear ribosomal DNA

PP Posterior probabilities

Q Length to width ratio

RPB1 RNA polymerase II largest subunit

RPB2 RNA polymerase II second largest subunit

TEF1 Translation elongation factor 1-alpha

UNIPAR Mycological herbarium of the University of Parakou

## Results

### Phylogenetic analyses of sequences of *Trametes* species from Benin

**ITS dataset.** The 25 ITS sequences obtained from *Trametes* spp. from Benin clustered in eight distinct clades (Suppl. material 2). All sequences of *Trametes* spp. from Benin fell into the monophyletic corresponding clades except the clade of *Trametes lactinea* (Berk.) Sacc., which, besides sequences of *T. lactinea*, accommodated also sequences of *Trametes cubensis* (Mont.) Sacc. with a very high support (BP = 1.00/BS = 100). Sequences of specimens of *Trametes* sp. (OAB0022 and OAB0023) from Benin formed a separated and well-supported clade within the *Trametes* clade (BP = 0.73/BS = 66).

**Figure 3.**
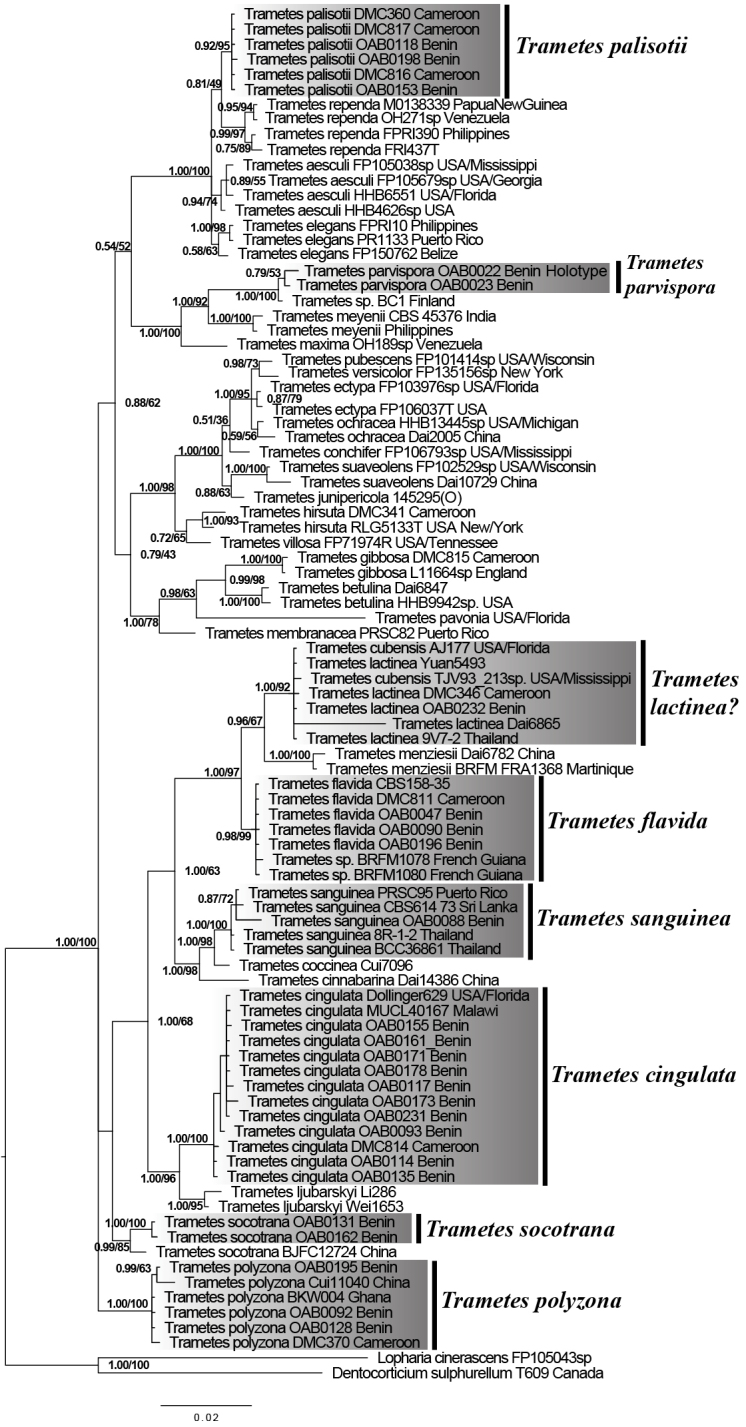
ML phylogeny of *Trametes* spp. based on combined ITS-LSU dataset. Branch support values given as PP/BS. All clades where newly generated sequences clustered are highlighted in grey and bars with names are given beside for more readability. Taxon names are followed by voucher or stain number and country of origin.

**Figure 4.**
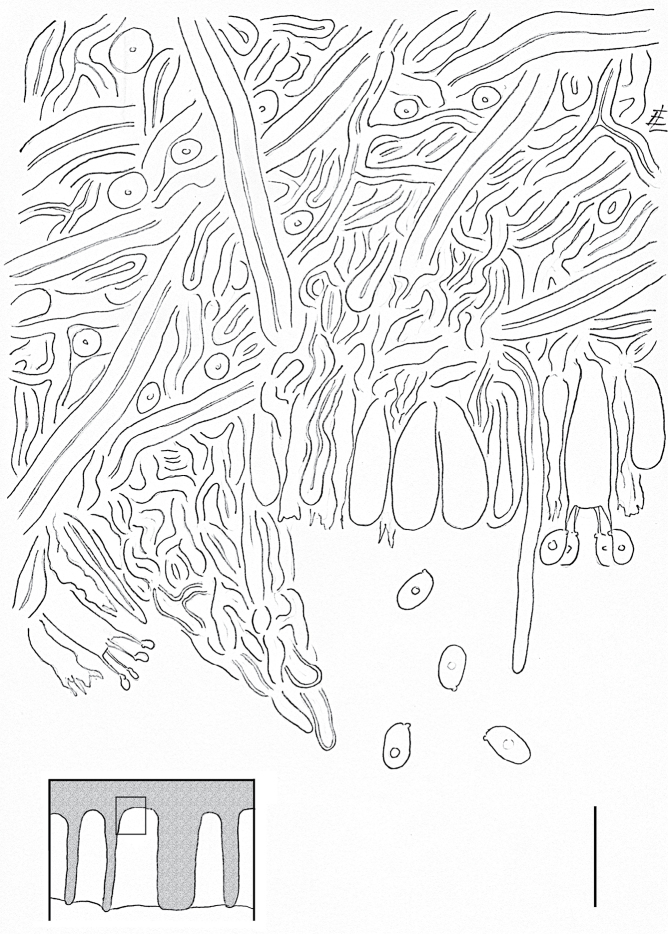
Crossection of the hymenium at the base of a pore of *Trametes parvispora*. Basidiospores, hyphae, basidia, basidioles, and a hyphal peg are showing. The box (lower left corner) shows the location (small rectangle) of the line drawing in the cross-section of the hymenophore. Scale bar = 10 μm

**ITS-LSU dataset.** Results of ML and of BY show higher congruency, higher support values, and a higher number of resolved nodes than the results obtained with ITS data only. As evident by the ITS dataset, the sequence of *T. lactinea* from Benin clustered in addition to other sequences of *T. lactinea* retrieved from GenBank with sequences of *T. cubensis* with high support (BP = 1.00/BS = 92). Like in the analysis of the ITS dataset, sequences of *Trametes* sp. from Benin formed a distinct clade (Fig. 3). The two sequences of the new species of *Trametes* from Benin clustered in a distinct lineage within the *Trametes* clade (Figs 2I, J; 4). The clade of the *T. elegans* species complex is presented in the section below.

### Phylogenetic placement of *Trametes elegans* from tropical Africa within the *Trametes elegans* species complex

**Figure 5.**
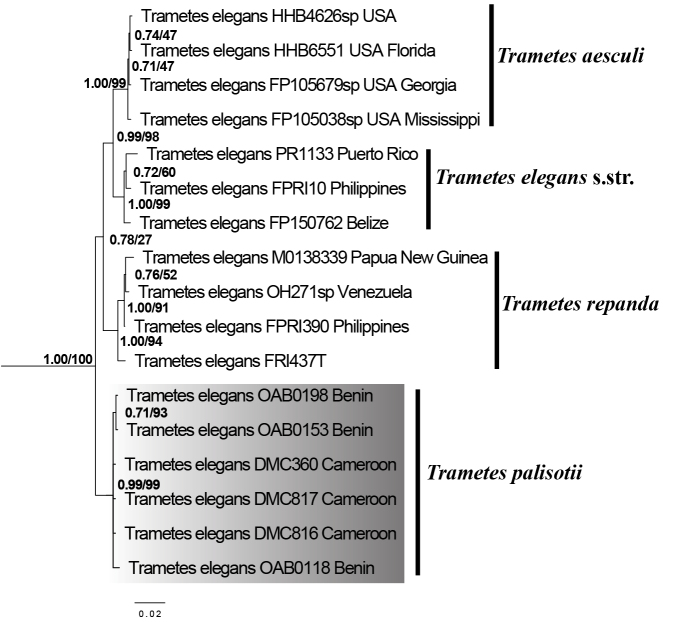
ML phylogeny of *Trametes elegans* species complex based on combined dataset of four-gene regions (ITS, RPB1, RPB2, TEF1). Branch support values given as PP/BS. Sequences of *T. elegans* from tropical Africa investigated in this study are highlighted in grey.

The phylogenetic trees generated from individual gene regions ITS, RPB1, RPB2, and TEF1 (Suppl. material 3) and the combined datasets (Fig. 5) show similar results for phylogenetic relationships within the *T. elegans* species complex. Four distinct and well-supported clades were evident in all datasets. The clade highlighted in grey (Fig. 5; Suppl. material 3) is distinct from all other clades within *T. elegans* species complex and highly supported in all individual gene and combined dataset. This clade contains only sequences of *T. elegans* from Benin and Cameroon.

### Taxonomy

#### Trametes parvispora

Taxon classificationFungiPolyporalesPolyporaceae

Olou, Yorou & Langer sp. nov.

4584EE87-6952-5F60-A0DB-93313708313D

830395

Figures 2I, J , 4

##### Diagnosis.

*Trametes parvispora* differs from known species of *Trametes* in the combination of the following characteristics: daedaleoid hymenophore, context whitish, thin 1–1.5 mm, homogeneous, without black lines, small spores 3.2–4.6 × 2.1–2.8 μm, regular hyphal pegs 25–30 μm long, cystidia absent, abundance of basidioles, and basidia 12–15 × 3–5 μm.

##### Type.

BENIN. Atlantic province, dry dense forest of Pahou in Ouidah, 6°23'2.97"N, 2°9'15.90"E, altitude: 33.1 m, on dead part of living tree of *Dialium guineense* Willd., leg. Boris A. Olou, sampling date: 21.07.2017, OAB0022 (dried specimen, holotype in UNIPAR and isotype in KAS). Holotype Sequences: ITSMK736989, LSUMK736964, and RPB2MN127965

##### Etymology.

*parvispora* (Lat.): referring to the small size of the spores.

##### Description.

Basidiomata probably perennial, sessile, pileate, applanate, semicircular, up to 13 cm long and 8 cm wide, up to 2.5 cm thick at the base, coriaceous to woody and hard when dry, without odour or taste when fresh. Pileus surface dull, glabrous, and whitish, zonate, margin thick, obtuse. Pore surface whitish, daedaleoid. Context whitish, thin (1–1.5 mm), homogeneous, without black lines.

Hyphal system trimitic, generative hyphae hyaline branched with clamp connections, thin-walled, 1.5–2.0 μm in diameter, acyanophilous; skeletal hyphae solid to thick-walled, hyaline, non-septate, 3–4 μm in diameter, totally dominating in the context, acyanophilous, tissues unchanged in KOH, unbranched; binding hyphae very common in both the context and trama, hyaline, thick-walled, acyanophilous, and much branched.

Cystidia absent, but the branches of the binding hyphae may easily be mistaken for thick-walled cystidia in the hymenium unless a careful examination is undertaken. Hyphal pegs present, especially at the base of pores, and regular, 25–30 μm long.

Basidia 12–15 × 3–5 μm, clavate, tetrasterigmatic, sterigmata 3 μm long; Basidioles numerous, similar in shape to basidia but slightly smaller than basidia, up to 4 μm in diameter.

Basidiospores broadly ellipsoid, hyaline, thin-walled, smooth, usually with one guttule each, negative in Melzer’s reagent, acyanophilous, (2.9)3.2–4.6(4.9) × 2.1–2.8(2.9) μm, L = 3.88 μm, W = 2.48 μm; Q = (1.17)1.24–1.91(2.05), Q = 1.57.

##### Ecology and distribution.

Saprotrophic, on dead part of living tree *Dialium guineense* and only known from dry dense forest of Pahou in southern Benin.

##### Additional materials examined.

BENIN. Atlantic province, dry dense forest of Pahou/ Ouidah, leg. Boris A. Olou, on dead wood of *D. guineense*, 21.07.2017, 6°23'3.07"N, 2°9'16.32"E, altitude 18.4 m a.s.l., OAB0023 (UNIPAR); on dead part of living tree of *D. guineense*, 6°23'2.49"N, 2°9'16.27"E, altitude 33.1 m a.s.l., 20.07.2018, OAB0267 (UNIPAR); at the same locality, 26.09.2018, OAB0268 (UNIPAR).

## Discussion

### *Trametes* spp. diversity in Benin

In Benin, seven species of *Trametes* were previously reported (Olou et al. 2019). By the present, study two additional species, namely *T. lactinea* and Trametes aff. versicolor (Fig. 2E, F, N), were recorded in addition to previous species. Thus, to our knowledge, nine species of *Trametes* are currently known for Benin. Of these nine species, only two species, *T. elegans* and *T. sanguinea*, were reported in Benin until 2002 (Yorou and De Kesel 2002). The remaining seven species, namely *T. cingulata*, *T. flavida*, *T. lactinea*, *T. parvispora*, *T. polyzona*, *T. socotrana*, and Trametes aff. versicolor, were recorded between 2017 and 2018. Given this history, it is most likely that more species will be found. Nonetheless, this number is significant when compared to the total diversity of 9–14 species of *Trametes* reported for Europe (Ryvarden and Gilbertson 1994; Ryvarden and Melo 2014). Further studies are needed to document the overall diversity of species of *Trametes* in Benin.

### Phylogenetic positions of *Trametes* species of Benin

To place specimens of *Trametes* spp. from Benin in a larger phylogenetic context, we generated sequences of several genes. Generated sequences were placed into the phylogeny of the genus *Trametes* as established by Justo and Hibbett (2011). Eight distinct clades corresponding to eight different species were obtained from these sequences.

Our phylogenetic analyses from ITS and combined ITS-LSU datasets reveal sequence similarities and taxonomic misplacement within the clades of *T. flavida* and *T. lactinea* (Fig. 3; Suppl. material 2). The clade of *T. flavida* accommodated, in addition to sequences of *T. flavida*, sequences of *Trametes* sp. from French Guiana which is known as *Leiotrametes* sp. (Welti et al. 2012). This species was proposed as a new species by Welti et al. (2012). Here, *Trametes* sp. clustered together with *T. flavida* with high support in the ITS dataset (PP = 0.84/BS = 89) and the combined ITS-LSU datasets (PP = 0.98/BS = 99). Both species share also high morphological similarity (Welti et al. 2012; Fig. 2C, D) and a tropical distribution. We therefore suggest that *Trametes* sp. from French Guiana should not be considered as a new species but should be referred to as *T. flavida*. In addition to the *T. flavida* clade, our phylogenetic analyses showed that the *T. lactinea* clade contains not only sequences of *T. lactinea*, but also sequences of *T. cubensis* with high support in the ITS and ITS-LSU datasets (Fig. 3; Suppl. material 2). This result is similar to previous phylogenetic analyses on *Trametes* using the ITS marker (Justo and Hibbett 2011; Carlson et al. 2014). *Trametes lactinea* and *T. cubensis* are still valid names and both species share quite similar morphological characters. They are characterized by an applanate, broadly attached to dimidiate, white to cream basidiomata and a white to cream pore surface (Ryvarden and Johansen 1980; Gilbertson and Ryvarden 1987). Nevertheless, although both species are sharing quite similar morphological characters, they also differ in some characters. *Trametes. cubensis* is characterized by an annual basidioma, small pores, almost invisible to the naked eye, 5–7 per mm, and cylindrical basidiospores 7–9 × 3–3.5 μm (Gilbertson and Ryvarden 1987), while *T. lactinea* has an annual to perennial basidioma and large pores, which are visible to the naked eye, mostly 1.5–2 per mm, but can reach up to 3–4 (5) per mm in some specimens with cylindrical-ellipsoid basidiospores 4–7.5 × 2.2–3 μm (Ryvarden and Johansen 1980). Our specimen of *T. lactinea* (Fig. 2E, F) matches the morphological description of *T. lactinea* with 3–4 pores per mm, but we did not observe any spore despite numerous attempts. Thus, considering the result of our phylogenetic analyses, absence of spores in our *T. lactinea* specimen, and the high morphological similarity between species within *Trametes* (Gilbertson and Ryvarden 1987), we cannot reasonably distinguish *T. lactinea* from *T. cubensis*. Further morphological, chemotaxonomic, and molecular studies integrating proteins coding genes (e.g. RPB1, RPB2, and TEF1) are therefore needed to confirm whether *T. lactinea* and *T. cubensis* refer to the same species.

Previously the phylogenetic resolution of *T. cingulata* was problematic due to low sequence availability. Here we generated a total of 17 de novo sequences and show that *T. cingulata* appears as a monophyletic group within *Trametes* with high support in ITS and combined ITS-LSU datasets respectively (PP = 1.00/BS = 97) and (PP = 1.00/BS = 100) (Fig. 3; Suppl. material 2). Thus, contrary to the uncertain position of *T. cingulata* within the genus *Trametes* (Welti et al. 2012), our results revealed that the latter does not belong to *Trametes* sensu stricto in the sense of Justo and Hibbett (2011) and Welti et al. (2012) (Fig. 3; Suppl. material 2) but rather to *Trametes* sensu lato.

### Species diversity in the *Trametes elegans* species complex

The specimens from Benin identified as members of the *T. elegans* species complex correspond to the morphological descriptions of *T. elegans* by Gilbertson and Ryvarden (1987) and Ryvarden and Johansen (1980). The clades evident in all datasets within the *T. elegans* complex (Figs 3, 5; Suppl. material 2, 3) represent three clades previously attributed to three different species by Carlson et al. (2014), and a new clade highlighted in grey (Fig. 5; Suppl. material 3) represents specimens of *T. elegans* from Benin and Cameroon (Tropical Africa). This new clade contains only sequences of *T. elegans* from Benin and Cameroon due to the non-publication of most *T. elegans* sequences from tropical Africa (Olusegun 2015; Awala and Oyetayo 2016; Ueitele et al. 2018). Thus, prior to this study, only sequences of *T. elegans* from Cameroon and Gabon are available in GenBank for Africa. However, the sequences of *T. elegans* from Gabon (GenBank accession number: KY449397, KY449398) were not considered because they fell outside the *T. elegans* species complex and were instead closely related to *T. lactinea*. We, therefore, excluded these sequences from our analyses. All in all, since the sequences of *T. elegans* from tropical Africa investigated in this study are demarcated from sequences of *T. elegans* s. str., the adoption of another correct name for specimens of *T. elegans* from this area is necessary.

Specimens belonging to the *T. elegans* species complex have been reported in the past for tropical African countries (Ryvarden and Johansen 1980), with the first name applied to such specimens being *Daedalea amanitoides* P. Beauv., which was based on a specimen from Nigeria (cited as kingdom of Oware) (Palisot-Beauvois 1804). The morphological characteristics evident in the very short description and illustration of a fruiting body of *D. amanitoides* match the characteristics of the specimens examined in this study. However, for reasons that we ignore, Fries (1821) replaced this name (*D. amanitoides)* by the name *Daedalea palisotii* Fr., which is sanctioned and therefore must be used. The combination *Trametes palisotii* (Fr.) Imazeki (Imazeki 1952) is available and must be used for African specimens known previously as *T. elegans* (Fig. 5).

### Phylogenetic position and taxonomy of the new species *Trametes parvispora*

The sequences belonging to the new species named *T. parvispora* form a distinct and well-supported clade in the ITS and the combined ITS-LSU datasets (Fig. 3; Suppl. material 2). This species forms a sister clade with the still formally undescribed *Trametes* sp. (KT896651) from Finland. However, unlike *T. parvispora* where fruiting bodies were available for morphological characterization (Fig. 2I, J), the Finnish specimen was isolated as mycelium from the bark beetle *Ips typographus* L. (Linnakoski et al. 2016). Thus, anatomical and morphological comparisons are currently not possible. Furthermore, both sequences of *T. parvispora* share a clade with *Trametes meyenii* (Klotzsch) Lloyd. This clade was confirmed by phylogenetic analyses including two additional markers RPB1 and RPB2 (Suppl. material 4). *Trametes meyenii* has hispid and cream-yellow pilei, irpicoid and white to ochraceous hymenophore, pores 1–3 per mm, 4.5–6 × 2–2.5 μm basidiospores (Zmitrovich et al. 2012), whereas *T. parvispora* has glabrous and whitish pilei, a daedaleoid and white hymenophore, 3.2–4.6 × 2.1–2.8 μm basidiospores, and the presence of regular hyphal pegs (Figs 2I, J, 4). These morphological differences confirm that *T. parvispora* and *T. meyenii* are distinct species as shown by the phylogenetic analyses (Fig. 3; Suppl. material 2, 4). However, some species lacking DNA sequences, namely *Trametes barbulata* Corner, *Trametes daedaleoides* Corner, and *Trametes rugosituba* Corner (Corner 1989; Hattori 2005; Hattori and Sotome 2013), share with *T. parvispora* a quite similar spore size range. But the latter species differs from each other species by the combination of macro- and microscopic characteristics outlined above. Thus, the rare anatomic features of the regular hyphal pegs and the small size of the basidiospores together with the phylogenetic placement within the *Trametes* clade, provide enough evidence for *T. parvispora* as a distinct new species.

## Supplementary Material

XML Treatment for Trametes parvispora

## Supplementary materials

Supplementary material 1 Names, voucher numbers, and substrates of specimens of *Trametes* spp. collected in Benin Data type: species data https://binary.pensoft.net/file/388144This dataset is made available under the Open Database License (http://opendatacommons.org/licenses/odbl/1.0/). The Open Database License (ODbL) is a license agreement intended to allow users to freely share, modify, and use this Dataset while maintaining this same freedom for others, provided that the original source and author(s) are credited.Boris Armel Olou, Franz-Sebastian Krah, Meike Piepenbring, Nourou Soulemane Yorou, Ewald Langer

Supplementary material 2 ML phylogeny of *Trametes* spp. based on a single gene region ITS Data type: phylogeny data Explanation note: Support values are given as PP/BS. Newly generated sequences are highlighted in bold italic. Taxon names are followed by the voucher or stain numbers and the country of origin. https://binary.pensoft.net/file/388145This dataset is made available under the Open Database License (http://opendatacommons.org/licenses/odbl/1.0/). The Open Database License (ODbL) is a license agreement intended to allow users to freely share, modify, and use this Dataset while maintaining this same freedom for others, provided that the original source and author(s) are credited.Boris Armel Olou, Franz-Sebastian Krah, Meike Piepenbring, Nourou Soulemane Yorou, Ewald Langer

Supplementary material 3 ML phylogeny of *Trametes elegans* species complex as recovered from four individual gene regions Data type: phylogeny data Explanation note: Support values are given as PP/BS. Taxon names are followed by the voucher or stain numbers and the country of origin. The clade formed by the sequences of T. elegans from tropical Africa are highlighted in grey. https://binary.pensoft.net/file/388146This dataset is made available under the Open Database License (http://opendatacommons.org/licenses/odbl/1.0/). The Open Database License (ODbL) is a license agreement intended to allow users to freely share, modify, and use this Dataset while maintaining this same freedom for others, provided that the original source and author(s) are credited.Boris Armel Olou, Franz-Sebastian Krah, Meike Piepenbring, Nourou Soulemane Yorou, Ewald Langer

Supplementary material 4 ML phylogeny of *Trametes parvispora*, based on two-gene dataset (RPB1, RPB2) Data type: phylogeny data Explanation note: Support values given as PP/BS. Taxon names are followed by the voucher or stain numbers and the country of origin. https://binary.pensoft.net/file/388147This dataset is made available under the Open Database License (http://opendatacommons.org/licenses/odbl/1.0/). The Open Database License (ODbL) is a license agreement intended to allow users to freely share, modify, and use this Dataset while maintaining this same freedom for others, provided that the original source and author(s) are credited.Boris Armel Olou, Franz-Sebastian Krah, Meike Piepenbring, Nourou Soulemane Yorou, Ewald Langer
