# Supplementary material for: Diversity of Trametes (Polyporales, Basidiomycota) in tropical Benin and description of new species Trametes parvispora
Source: MycoKeys. 2020 Mar 10;65:25–47. doi: 10.3897/mycokeys.65.47574 (PMC7078339; doi:10.3897/mycokeys.65.47574)
Supplement: Supplementary material 1 [file mycokeys-65-025-s004.pdf]

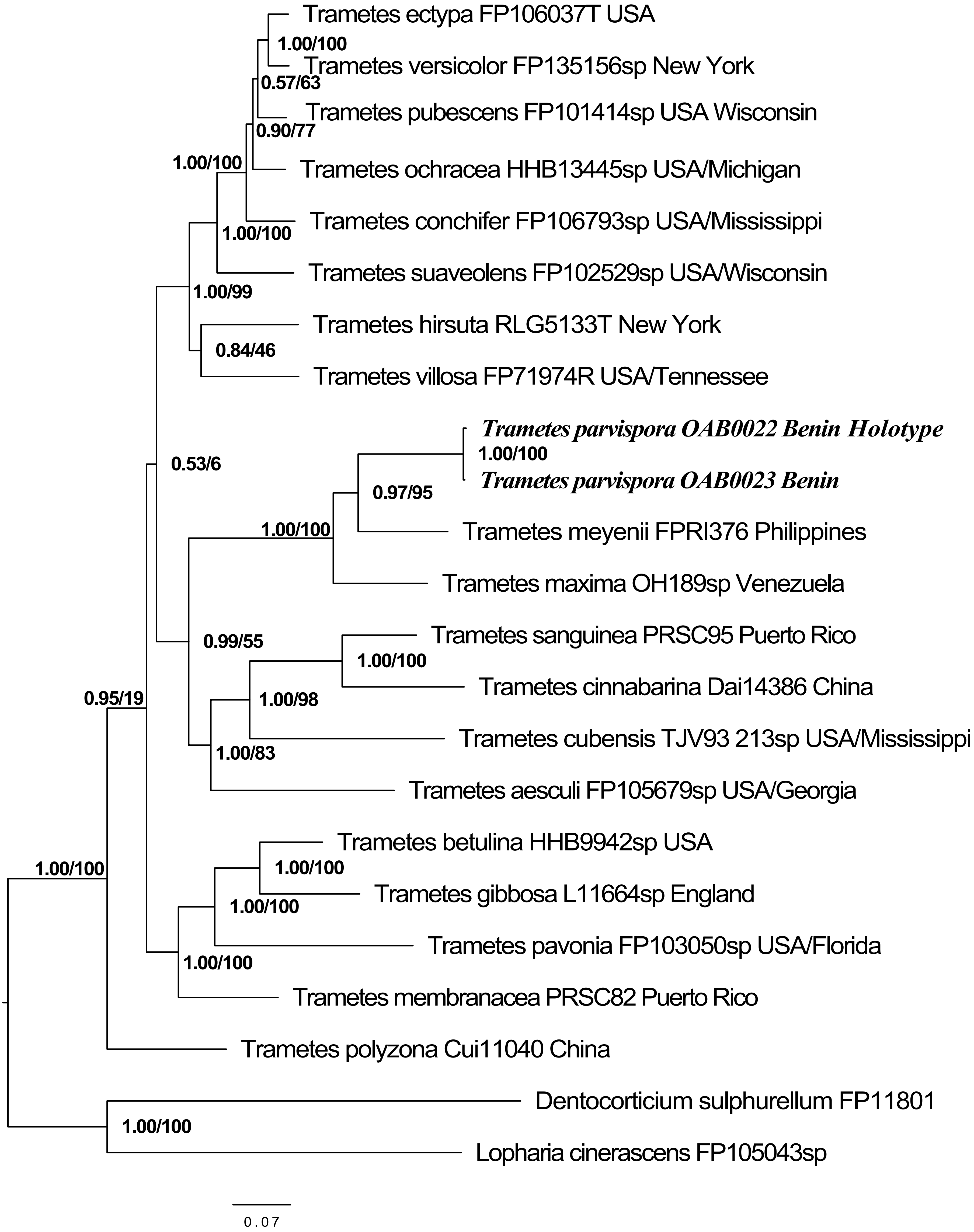

Supplementary file 4. ML phylogeny of Trametes parvispora, based on two-gene dataset (RPB1, RPB2). Support values given as PP/BS. Taxon names are followed by the voucher or stain numbers and the country of origin.
